# Induction of endoplasmic reticulum stress and unfolded protein response constitutes a pathogenic strategy of group A streptococcus

**DOI:** 10.3389/fcimb.2014.00105

**Published:** 2014-08-04

**Authors:** Moshe Baruch, Baruch B. Hertzog, Miriam Ravins, Aparna Anand, Cheng Catherine Youting, Debabrata Biswas, Boaz Tirosh, Emanuel Hanski

**Affiliations:** ^1^Department of Microbiology and Molecular Genetics, Faculty of Medicine, The Hebrew University of Jerusalem (HUJI)Jerusalem, Israel; ^2^Department of Microbiology, Center for Research Excellence and Technological Enterprise (CREATE), National University of Singapore (NUS) and NUS-HUJISingapore; ^3^The School of Pharmacy, Institute for Drug Research, The Hebrew University of JerusalemJerusalem, Israel

**Keywords:** group A streptococcus, UPR, metabolism, virulence, asparagine, asparaginase

## Abstract

The connection between bacterial pathogens and unfolded protein response (UPR) is poorly explored. In this review we highlight the evidence showing that group A streptococcus (GAS) induces endoplasmic reticulum (ER) stress and UPR through which it captures the amino acid asparagine (ASN) from the host. GAS acts extracellularly and during adherence to host cells it delivers the hemolysin toxins; streptolysin O (SLO) and streptolysin S (SLS). By poorly understood pathways, these toxins trigger UPR leading to the induction of the transcriptional regulator ATF4 and consequently to the upregulation of asparagine synthetase (ASNS) transcription leading to production and release of ASN. GAS senses ASN and alters gene expression profile accordingly, and increases the rate of multiplication. We suggest that induction of UPR by GAS and by other bacterial pathogens represent means through which bacterial pathogens gain nutrients from the host, obviating the need to become internalized or inflict irreversible cell damage.

The endoplasmic reticulum (ER) is an essential organelle that controls protein and lipid biosynthesis, protein folding and trafficking and calcium homeostasis (Berridge, 2002). Different perturbations at the cellular level can affect ER homeostasis inducing the accumulation of misfolded proteins within the ER lumen or changing its lipid composition. These processes ultimately lead to ER stress. To alleviate these conditions, the ER launches the unfolded protein response (UPR), allowing the cells to adapt to the environmental stresses and survive (Walter and Ron, 2011). However, under prolonged stress conditions, when stresses remain unmitigated, UPR triggers programmed cell death (Tabas and Ron, 2011; Woehlbier and Hetz, 2011).

In mammalian cells UPR is mediated by three major signal transduction pathways: PKR-like endoplasmic reticulum kinase (PERK), inositol-requiring enzyme 1 (IRE1), and activating transcription factor 6 (ATF6) (Figure 1). These signaling pathways are all initiated when misfolded proteins are sensed in the ER lumen. These three pathways combat ER stress through complementary strategies including: (a) attenuation of global protein translation to reduce the influx of client proteins into the ER; (b) up-regulation of chaperones and enzymes involved in refolding of misfolded proteins; and (c) enhancing ER-associated degradation (ERAD) to facilitate clearance of misfolded proteins from the ER (Schroder and Kaufman, 2005; Ron and Walter, 2007). As mentioned above, a variety of external stimuli have been shown to cause UPR. This includes abiotic stresses; pharmacological agents and toxins producing imbalance of ER calcium and redox; anti-inflammatory agents causing vigorous protein synthesis; energy deprivation, amino acids and ATP depletion, genetic mutations occurring in protein misfolding diseases and microbial pathogens (Schroder and Kaufman, 2005; Yoshida, 2007; Walter and Ron, 2011). Since there are several potential cross-talks between UPR and microbial sensing pathways that trigger immune responses (Hotamisligil, 2010; Hasnain et al., 2012; Hetz, 2012; Martinon, 2012; Claudio et al., 2013), investigators started to explore how microbial pathogens cope with UPR that they induce and whether or not they are able to exploit UPR for their own “benefit” (Roy et al., 2006).

**Figure 1:**
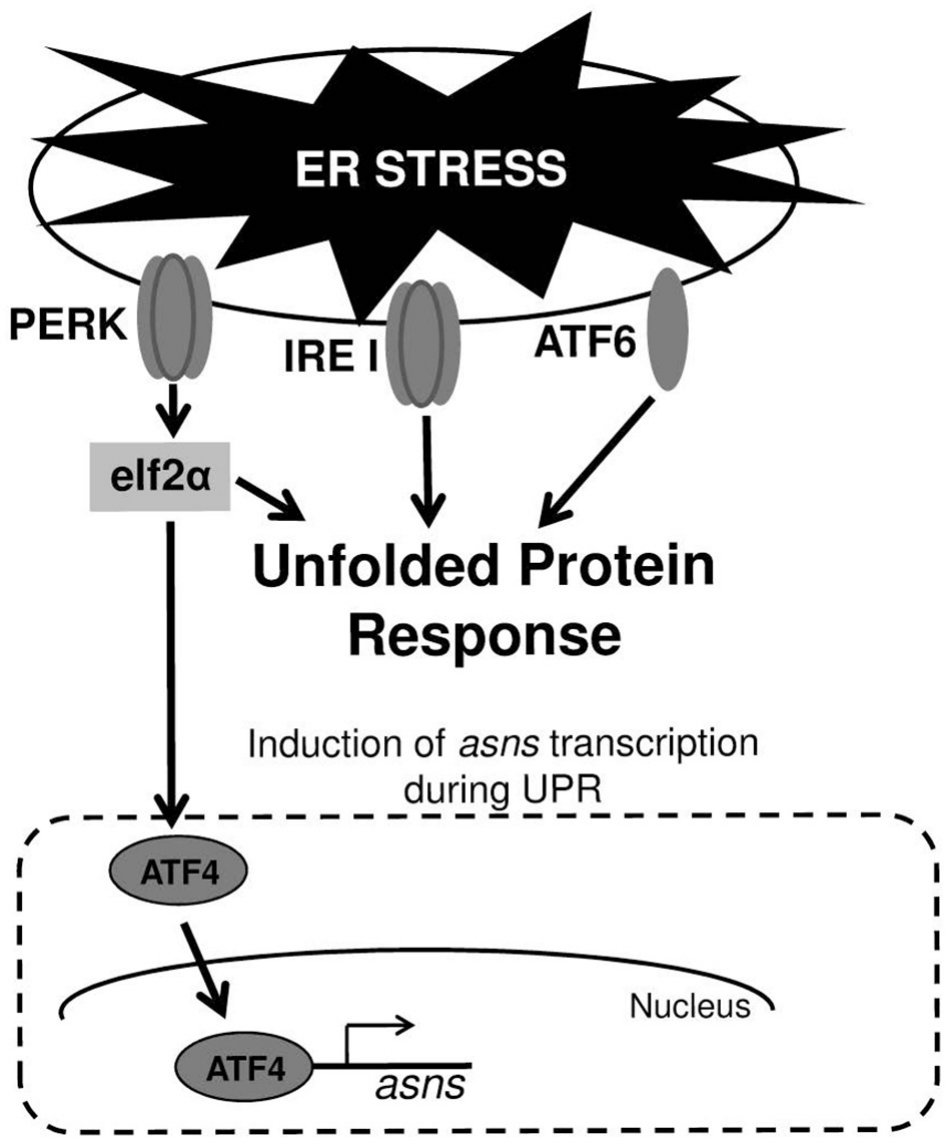
**ER stress and UPR. Upper panel**—the three transmembrane receptor proteins that are responsible for triggering UPR in cells experiencing ER stress. **Lower panel**—induction of *asns* transcription through the PERK/eIF2/ATF4 pathway. During UPR, PERK phosphorylates elf2α, which in turn elevates the translation of the transcription factor ATF4. ATF4 upregulates the transcription of several genes including that of *asns*.

Under viral infection these questions seem most relevant as viruses cause both cell stress through their replication and by over-expressing viral proteins (Zhang and Wang, 2012; Claudio et al., 2013; Hare and Mossman, 2013). Indeed, many viruses have evolved strategies to ensure completion of their infectious cycle by actively interfering with host translation shut-down and prolonging infected-cells life span, despite severe stress conditions (He, 2006; Roy et al., 2006; Martinon, 2012). Moreover, viruses developed strategies to differentially regulate the three UPR signaling pathways (Figure 1) and to dampen downstream inflammatory responses, and thus exploit ER stress for their own benefits (He, 2006; Roy et al., 2006; Martinon, 2012; Stahl et al., 2013).

Much less is known about the role of UPR in bacterial infections. Indeed, it was reported that the pathogen itself as well as bacterial toxins both induce UPR. Yet, it remained elusive whether UPR induction represents a genuine pathogenic trait of the bacteria, constitutes a pathway through which the host mounts immune defenses or is just a vicissitude of the interaction. For example, shiga toxins (Stxs) are genetically and structurally related cytotoxins expressed by the enteric pathogens *Shigella dysenteriae* serotype 1 and an expanding number of Shiga toxin-producing *Escherichia coli.* Following retrograde transport Stxs are translocated into the ER lumen and then the active fragment is translocated across the ER membrane to reach the cytoplasm where it de-purinates the 28S rRNA subunit of the ribosome. This in turn, triggers UPR and leads to downstream signaling through the p38 mitogen-activated protein kinases (MAPK) cascades (Liang et al., 2006), which appear to be critical for activation of innate immunity and regulation of apoptosis (Tesh, 2012). Cholera toxin (CT) is a major virulence factor of *Vibrio cholera* that reaches the lumen of the ER in a similar way to that of Stxs (Sandvig et al., 1992). In the ER lumen, CT unfolds and the A1 chain interacts with IRE1 to initiate UPR. The unfolded A1 chain co-opts the ER to retro-transport itself by the ERAD machinery into the cytosol, where it refolds, escapes degradation and becomes catalytically active. In addition, an inflammatory response is generated by the activated IRE1 RNase. This RNase degrades cellular RNAs that are detected by the retinoic-acid inducible gene 1 (RIG-1), a cytosolic sensor of RNA viruses. This in turn activates the NF-κ B and interferon pathways (Cho et al., 2013).

The ability to induce UPR is not limited only to Stxs and CT, but also exists for pore-forming toxins (PFTs) that constitute the largest class of bacterial toxins and are produced by the most clinically important bacterial pathogens. In *Caenorhabditis elegans* infected with bacteria expressing PFTs, UPR is induced and lose of ATF6 and IRE1 pathways (Figure 1) by genetic manipulations leads to hypersensitivity of the nematode to attack by PFT-producing bacteria. These findings suggest that ER homeostasis or induction of immune response via ER-signaling protects the host against these toxins (Bischof et al., 2008). *Brucella melitensis* is a facultative intracellular bacterium that fuses with the ER to replicate. This results in a marked reorganization of the ER around the replicating bacteria and triggering of UPR. UPR induction requires both live bacteria and the expression of a specific *Brucella* protein (Smith et al., 2013). Another facultative intracellular pathogen, *Listeria monocytogenes*, induces ER expansion and UPR prior to its entry into host cells. Its mutant, deficient of the PFT, listeriolysin O (LLO), is unable to induce UPR. Furthermore, induction of UPR by ER-stressors before infection with *L. monocytogenes* reduces bacterial intracellular loads, suggesting that UPR may represent a defense response of the host against *L. monocytogenes* infection (Pillich et al., 2012). The first indication that UPR induction by a bacterial pathogen could be a virulence strategy was reported for GAS. Cywes-Bentley and colleagues demonstrated that infection of keratinocyte by GAS deregulates intracellular calcium through the action of the PFT, protein- SLO. This in turn causes UPR, subsequently leading to loss of epithelial integrity, cell detachment and apoptosis (Cywes Bentley et al., 2005).

GAS is an obligate human pathogen and the fourth most common bacterial cause of human mortality (Carapetis et al., 2005). GAS causes a vast array of human manifestations ranging from mild infections such as pharyngitis and impetigo to highly invasive and life-threatening infections such as necrotizing fasciitis and toxic shock, as well as to the autoimmune syndromes rheumatic fever and glomerulonephritis (Cunningham, 2000; Walker et al., 2014). SLO and SLS are essential virulence factors of GAS as was demonstrated both in *ex-vivo* and *in-vivo* studies (Walker et al., 2014). SLO is a PFT belonging to the family of cholesterol-dependent cytolysins (CDCs) produced by several pathogenic Gram-positive bacteria including *Streptococcus, Clostridium*, and *Listeria* species. CDCs share many features including, a similar overall molecular structure, mechanisms of membrane recognition and pore formation (Hotze and Tweten, 2012). SLO is co-expressed with GAS NAD-glycohydrolase (SPN) and SLO-mediated translocation of SPN has been shown to be an additional way by which this toxin contributes to GAS virulence (Madden et al., 2001; Bricker et al., 2002). Another toxin with which SLO acts in concert during GAS infections is SLS (Ginsburg and Kohen, 1995; Fontaine et al., 2003; Watanabe et al., 2013). SLS is a small, ribosomally produced bacteriocin-like toxin that undergoes heterocyclic modifications at specific residues to confer activity. As SLO, SLS-like peptides are produced by some streptococci and other Gram-positive pathogens as *Clostridia, Listeria*, and *Staphylococci* species (Molloy et al., 2011). Finally, both SLO and SLS are delivered into host cells more efficiently by adhering bacteria compared to non-adhering bacteria, thus close contact of the bacteria to the cell promotes efficient delivery of the toxins (Ofek et al., 1990; Ruiz et al., 1998).

Although GAS has been considered as an extracellular pathogen, studies from various laboratories have shown that the bacterium has the propensity to invade different cell types *in vitro* (Courtney et al., 2002). This capacity was suggested to contribute to GAS persistence within the host. Indeed, GAS was cultivable from surgical specimens of human tonsils, even after treatment of the excised tissue with antibiotics (Osterlund et al., 1997). Since GAS does not proliferate within mammalian cells, the significance of GAS intracellular phase was not explored in depth in *in vivo* models of human infections. However, internalization of GAS via the clathrin-dependent pathway was reported to be inhibited by low doses of SLO when the latter was produced extracellularly, whereas intracellularly produced SLO protected GAS from various modes of intracellular killing (Logsdon et al., 2011; O'Seaghdha and Wessels, 2013).

Recently it was discovered that GAS induces UPR to capture ASN from the host (Baruch et al., 2014). This was found while investigating the conditions under which the GAS quorum sensing locus *sil* is self-activated. *sil* is situated on an mobile genetic element that may have been acquired before GAS speciation and remained present in about 20% of GAS clinical isolates and is widely prevalent in the GAS genetically close relative *Streptococcus dysgalactiae* subsp. *equisimilis* (Belotserkovsky et al., 2009). In the GAS M14 serotype, *sil* controls virulence as was shown using different animal models of human NF (Hidalgo-Grass et al., 2002, 2004; Kizy and Neely, 2009). Hitherto, it was possible to activate *sil* by providing the bacterium with a minute quantity of the mature synthetic autoinducer peptide SilCR (Belotserkovsky et al., 2009), but the conditions under which *sil* is naturally self-activated were not identified. Later, it was discovered that *sil* is temporarily self-activated *in vivo*, during the initial stages of soft-tissue infection in a murine model of human NF. Furthermore, it was discovered that *sil* is also activated *ex vivo* upon adherence to various types of eukaryotic cells (Baruch et al., 2014).

Cyews-Bently et al. showed that GAS induces SLO-mediated ER-stress at low multiplicity of infection (MOI) of keratinocyte cells due to dysregulation of intracellular calcium (Cywes Bentley et al., 2005). In accordance with these findings, it was shown that *sil* activation occurred at low MOI of intact but not lysed eukaryotic cells, did not required internalization of GAS and was mediated by delivery of SLO and SLS toxins (Baruch et al., 2014). To delineate the cellular process that is triggered by SLO and SLS delivery the involvement of autophagy, apoptosis, and necrosis that are affected by the hemolysin toxins and were shown to be linked to GAS pathogenesis was examined (Baruch et al., 2014). Using mutated mouse embryonic fibroblast cells (MEFs) in combination with various pharmacological agents, the involvement of the indicated cellular processes in *sil* activation were ruled out. The facts that host cell intactness was essential to observe *sil* activation and that hemolysin toxins were involved, together with the report that SLO triggers ER stress (Cywes Bentley et al., 2005), hinted at the involvement of the latter process. Indeed, induction of UPR using the ER stressors thapsigargin (TG), and dithiothreitol (DTT) produced a conditioned media capable of activating *sil.* Furthermore, addition of TG to MEFs-infected by GAS accelerated *sil* activation (Baruch et al., 2014). During the testing of different eukaryotic cells for the ability to activate *sil*, it was discovered that ASN *per-se* is responsible for *sil* activation (Baruch et al., 2014). This finding together with the fact that *asns* transcription of host cells is strongly upregulated during UPR through the PERK-eIF2-ATF4 pathway (Figure 1) (Balasubramanian et al., 2013), led to the examination of *asns* transcription during MEFs infection by GAS. As predicted, it was found that there is a significant increase in *asns* transcription in GAS-infected MEFs that is dependent on SLO and SLS (Baruch et al., 2014). Taken together, these results supported the model in which delivery of SLO and SLS toxins from GAS to eukaryotic cells during GAS adherence generates ER stress. This in turn leads to UPR, enhanced production of the response regulator ATF4, activation of ASNS and release of ASN to the medium, through a mechanism yet unknown (Figure 2). ASN is than sensed by GAS to activate *sil*.

**Figure 2:**
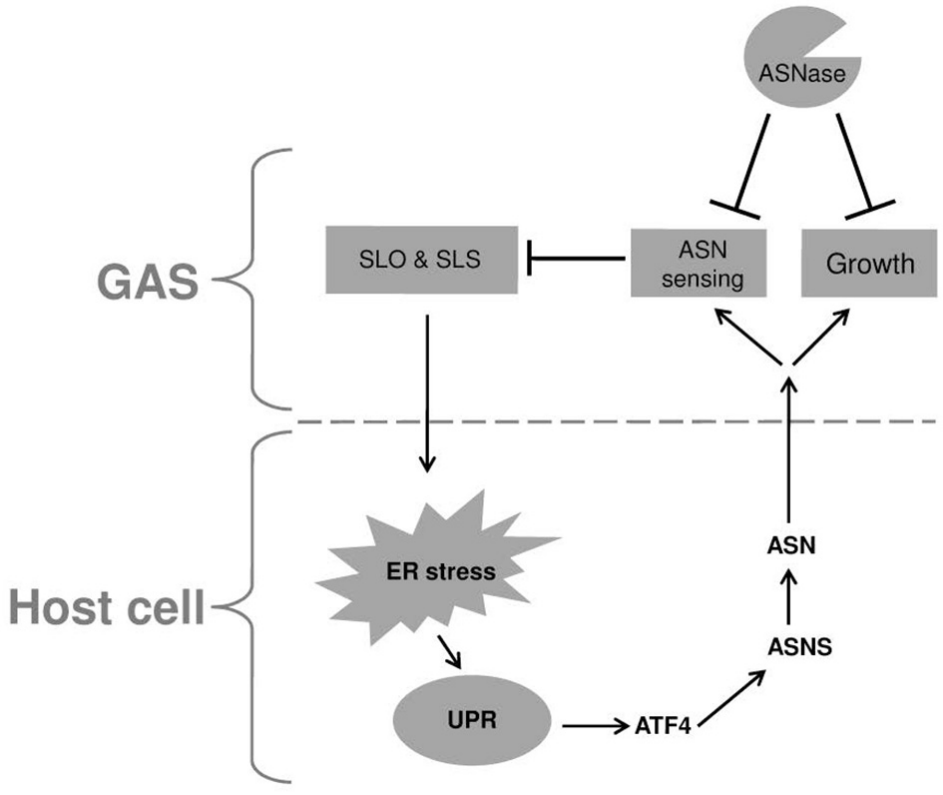
**A model describing the reciprocal relationship between GAS and infected host cell**. Upon adherence, GAS delivers the SLO and SLS toxins to the host cell. These toxins induce ER stress causing UPR, which in turn is responsible for the elevated transcription of *asns* gene through the PERK-eIF2-ATF4 pathway. The enhanced activity of ASNS leads to release of ASN to the culture medium which is sensed by GAS, resulting in reduced transcription of both SLO and SLS. In addition, GAS utilizes the released ASN to enhance its rate of proliferation. Both effects of host ASN on GAS can be inhibited by the addition of bacterial ASNase a widely used chemotherapeutic agent.

To corroborate this model, asparaginase (ASNase), which is widely used as a chemotherapeutic agent (Pui et al., 2008) was tested for its ability to inhibit *sil* activation. Indeed, ASNase obliterated *sil* activation *ex vivo* and *in vivo*, but most fascinatingly, also arrested GAS growth (Baruch et al., 2014). It was found that ASNase inhibits the growth of GAS irrespective of the serotype and presence or absence of *sil* (Figure 2). Therefore, it was decided to profile by RNA sequencing (RNA-seq) the RNA expression, in the globally disseminated highly invasive M1T1 GAS clone, (Maamary et al., 2012), after the addition of ASNase. It was found that 16.7% of GAS genes had a significantly altered expression in the absence vs. the presence of ASN (Baruch et al., 2014). Among others, the transcription of genes involved in GAS replication such as *polA* and *lig* was down regulated in ASN absence, while the transcription of genes encoding SLO and SLS were upragulated (Baruch et al., 2014 and Figure 2). Most importantly, testing the ability of ASNase to prevent GAS bacteremia showed that ASNase prevented GAS proliferation in whole human blood and in a murine model of human GAS bacteremia (Baruch et al., 2014).

It is suggested that the above-mentioned mechanism of GAS to gain ASN from the host is a central feature in its pathogenesis. This notion is supported by the findings that GAS involves SLO and SLS in this process. These two toxins are considered to constitute key virulence factors of GAS and are involved in many of GAS manifestations (Walker et al., 2014). Moreover, both SLO and SLS levels of transcription are strongly augmented by the absence of ASN (Baruch et al., 2014 and Figure 2). This finding suggests that ASN-mediated sensing of the host by GAS allows the bacterium to regulate the production of its main two toxins, in order to reach a level that on one hand permits GAS to stress the host but on the other hand to maintain homeostasis and avoid inflicting an irreversible damage. This trait is sustained mainly because GAS stresses the host ER, which has an intrinsic capacity to alleviate a wide range of stresses by mounting the UPR response (Schroder and Kaufman, 2005; Zhang and Kaufman, 2006; Ron and Walter, 2007; Walter and Ron, 2011).

The concentration of released ASN allows GAS to assess its population size that is in close contact with the host as well as the host stress status, i.e., whether or not it could sustain more stress to release even more nutrients without progressing into irreversible cell death. Consequently, these assessments may be used by GAS to regulate the level of virulence factors expression, and avoid their metabolically costly production before reaching a critical mass that ensures the successful mounting of invasive infection. Indeed it was found using the murine model of soft-tissue infection, that *sil*, which serves as a reporter for ASN sensing, is activated transiently at the very early steps of the infection, way before GAS disseminates into internal organs (Baruch et al., 2014).

It was observed that low levels of SLO inhibit GAS internalization by human keratinocytes (Logsdon et al., 2011). Intriguingly, it was reported that the facultative intracellular pathogen *L. monocytogenes* induces UPR through its LLO toxin (which shares 58.0% amino acids similarity with SLO) when present extracellularly. This in turn reduces the level of intracellular bacteria, due to the triggering of host defense responses (Pillich et al., 2012). It is speculated that *L. monocytogenes* regulates LLO expression also upon sensing of host metabolites that are released upon UPR induction. Finally, it was reported that *Mycobacterium tuberculosis*, which induces ER stress in granulomas during infection in humans (Seimon et al., 2010), exploits host ASN to assimilate nitrogen and resist acid stress during infection (Gouzy et al., 2014). Similar mechanisms of nitrogen assimilation were reported for *Helicobacter pylori* (Shibayama et al., 2011) and *Campylobacter jejuni* (Hofreuter et al., 2008). In summary, these studies emphasize the tight connection that has evolved during evolution between physiology and virulence. Understanding of this connection at the molecular level should pave the way for development of new ways to control severe human infectious diseases.

## Conflict of interest statement

The authors declare that the research was conducted in the absence of any commercial or financial relationships that could be construed as a potential conflict of interest.
